# Novel monosaccharide fermentation products in *Caldicellulosiruptor saccharolyticus* identified using NMR spectroscopy

**DOI:** 10.1186/1754-6834-6-47

**Published:** 2013-04-03

**Authors:** Nancy G Isern, Junfeng Xue, Jaya V Rao, John R Cort, Birgitte K Ahring

**Affiliations:** 1 Environmental Molecular Sciences Laboratory, Pacific Northwest National Laboratory, Richland, WA, 99354, USA; 2 Fundamental and Computational Sciences Directorate, Pacific Northwest National Laboratory, PO Box 999, MSIN: K8-98, Richland, WA, 99352, USA; 3 Center for Bioproducts and Bioenergy, Washington State University, 2710 Crimson Way, Richland, WA, 99354, USA

**Keywords:** *Caldicellulosiruptor saccharolyticus*, Nuclear magnetic resonance, Bioproducts, Acetoin, 2,3-Butanediol, Ethylene glycol

## Abstract

**Background:**

*Caldicellulosiruptor saccharolyticus* is a thermophilic, Gram-positive, non-spore forming, strictly anaerobic bacterium of interest in potential industrial applications, including the production of biofuels such as hydrogen or ethanol from lignocellulosic biomass through fermentation. High-resolution, solution-state nuclear magnetic resonance (NMR) spectroscopy is a useful method for the identification and quantification of metabolites that result from growth on different substrates. NMR allows facile resolution of isomeric (identical mass) constituents and does not destroy the sample.

**Results:**

Profiles of metabolites produced by the thermophilic cellulose-degrading bacterium *Caldicellulosiruptor saccharolyticus* DSM 8903 strain following growth on different monosaccharides (D-glucose, D-mannose, L-arabinose, D-arabinose, D-xylose, L-fucose, and D-fucose) as carbon sources revealed several unexpected fermentation products, suggesting novel metabolic capacities and unexplored metabolic pathways in this organism. Both ^1^H and ^13^C nuclear magnetic resonance (NMR) spectroscopy were used to determine intracellular and extracellular metabolite profiles. One dimensional ^1^H NMR spectral analysis was performed by curve fitting against spectral libraries provided in the Chenomx software; 2-D homonuclear and heteronuclear NMR experiments were conducted to further reduce uncertainties due to unassigned, overlapping, or poorly-resolved peaks. In addition to expected metabolites such as acetate, lactate, glycerol, and ethanol, several novel fermentation products were identified: ethylene glycol (from growth on D-arabinose), acetoin and 2,3-butanediol (from growth on D-glucose, L-arabinose, and D-xylose), and hydroxyacetone (from growth on D-mannose, L-arabinose, and D-xylose). Production of ethylene glycol from D-arabinose was particularly notable, with around 10% of the substrate carbon converted into this uncommon fermentation product.

**Conclusions:**

The present research shows that *C. saccharolyticus*, already of substantial interest due to its capability for biological ethanol and hydrogen production, has further metabolic potential for production of higher molecular weight compounds, such as acetoin and 2,3-butanediol, as well as hydroxyacetone and the uncommon fermentation product ethylene glycol. In addition, application of nuclear magnetic resonance (NMR) spectroscopy facilitates identification of novel metabolites, which is instrumental for production of desirable bioproducts from biomass through microbial fermentation.

## Background

*Caldicellulosiruptor saccharolyticus* is a thermophilic, Gram-positive, non-spore forming, strictly anaerobic bacterium of interest in potential industrial applications, including the production of biofuels such as hydrogen or ethanol from lignocellulosic biomass through fermentation [1-6]. *C. saccharolyticus* has a broad substrate range, and can grow on a variety of simple (e.g., glucose, mannose, xylose, and arabinose) or complex (e.g. cellulose, hemicelluloses) carbohydrates that are often associated with lignocellulosic biomass [7]. *C. saccharolyticus* produces native cellulases and hemicellulases, enabling it to efficiently hydrolyze complex carbohydrates and use the released monosaccharides as carbon and energy sources. In addition, *C. saccharolyticus* can grow on biomass that is either pretreated (e.g., *Miscanthus* hydrolysate, sugar beet juice, and paper sludge) or untreated (e.g. wheat straw, pine wood, and bagasse) [6]. Furthermore, *C. saccharolyticus* is able to co-ferment different monosaccharides, such as glucose and xylose, without exhibiting carbon catabolite repression [4,5], and it grows at high temperatures (optimum 65°C-70°C) and tolerates a broad temperature range.

Because *C. saccharolyticus* appears well-suited for production of biofuels, its metabolism has been extensively studied. Aside from hydrogen, acetate is the major fermentation product, and lactate and ethanol are also produced by mixed fermentation pathways [2,3]. *C. saccharolyticus* has been reported to exhibit increased production of lactate, a more reduced end-product, during the transition to stationary phase, which coincides with a drastic decrease in glucose consumption and acetate production [8]. Previous work has shown that the Embden-Meyerhof (EM) pathway is the main route for glycolysis *in C. saccharolyticus*, with a theoretical yield of 4 moles of H_2_ and 2 moles of acetate per mole of glucose [3]. Analysis of the genome sequence reveals the presence of all of the EM-pathway enzymes [4]. No evidence has been found for the presence of the Entner-Doudoroff (ED) and oxidative pentose phosphate pathways in *C. saccharolyticus*[3,4].

Identification of metabolites can provide insight into metabolic pathway utilization [9]. High-resolution, solution-state nuclear magnetic resonance (NMR) spectroscopy is a useful method for studying the changes in concentrations and fluxes of metabolites that result from growth on different substrates. NMR allows facile resolution of isomeric (identical mass) constituents and does not destroy the sample. To characterize the monosaccharide metabolism of *C. saccharolyticus* with the aim of characterizing its full metabolic potential for production of bioproducts, the present study used 1-D ^1^H-detected NMR spectroscopy together with resonance peak assignment and curve fitting for metabolite identification and quantification. Deconvolution and curve fitting in the Chenomx software package [10,11] have previously been used to determine metabolite profiles in a variety of microbial metabolomics applications [12-15], and these methods were utilized for the purposes of this study. To confirm the identification and for manual assignment of unidentified metabolites, 1-D ^13^C-detected and 2-D homo- and heteronuclear NMR techniques were used to analyze the metabolic profiles of *C. saccharolyticus* grown on various monosaccharides. Several novel fermentation products were identified and quantified, indicating novel metabolic capacities that are not predicted in the current understanding of metabolism implied by the genome of this thermophilic bacterium and suggesting new potentials for use of this organism in production of bioproducts from cellulosic biomass.

## Results and discussion

### Peak assignment using the Chenomx software

Metabolite profile analyses of the culture supernatants and extracts were compared with spectra of uninoculated growth medium to identify compounds produced and secreted by *C. saccharolyticus*. Across all of our studies, approximately 50 metabolites were identified and quantified using spectral deconvolution and library-based assignment routines in the Chenomx 7.61 software (Table 1, Table 2, and Additional file 1). Approximately twenty spectral features, many of them having low intensity, remained unassigned. However, two of the more prominent unassigned features were assigned using 2-D NMR spectroscopy to 2,3-butanediol and hydroxyacetone. These assignments were confirmed by comparison to prepared standards, and approximate concentrations were estimated using spectral deconvolution to estimate peak areas. The major reduced fermentation products of interest produced from growth on each monosaccharide are summarized in Table 2. Products such as amino acids that were also components of the growth media are not included, though studies with ^13^C-labeled glucose showed evidence for incorporation of ^13^C into some amino acids, notably alanine and glycine. All concentrations were determined using Chenomx except those so noted in Table 2. We have determined that concentrations of major metabolites quantified using Chenomx are accurate to within a few percent of the measured value for any particular sample (data not shown).

**Table 1:** **Observed**^**1**^**H and**^**13**^**C chemical shifts and corresponding assignments for major metabolites**

**Compound**	**Chemical shifts**					
acetate	184.1 (C1)	1.90 (H2)	25.9 (C2)			
acetoin (3-hydroxybutanone)	2.21 (H1)	27.8 (C1)	218.0 (C2)	75.6 (C3)	1.37 (H4)	21.1 (C4)
(RR/SS) 2,3-butanediol	1.14 (H1)	20.4 (C1)	3.61 (H2)	74.3 (C2)		
ethanol	3.66 (H1)	60.3 (C1)	1.17 (H2)	19.6 (C2)		
ethylene glycol	3.66 (H1)	65.3 (C1)				
glycerol	3.65 (H1a)	3.55 (H1b)	65.2 (C1)	3.78 (H2)	74.8 (C2)	
hydroxyacetone (1-hydroxypropanone)	4.37 (H1)	70.0 (C1)	215.3 (C2)	2.15 (H3)	27.2 (C3)	
lactate	185.2 (C1)	4.11 (H2)	70.8 (C2)	1.33 (H3)	22.7 (C3)	
propylene glycol (1,2-propanediol)	3.54 (H1a) 3.43 (H1b)	69.3 (C1)	3.87 (H2)	70.6 (C2)	1.13 (H3)	20.7 (C3)

Multiplicities in 1-D ^1^H spectra and observation of the expected cross peaks in 2-D COSY, HSQC, and HMBC spectra confirmed these assignments. All shifts are in agreement with expected and previously reported values.

**Table 2:** Major fermentation products in the batch culture supernatants as a function of sugar substrate

**Monosaccharide**	**Acetate**^**a**^	**Lactate**^**a**^	**Ethanol**	**Ethylene glycol**	**1,2-propanediol**^**b**^	**Glycerol**^**a**^	**Acetoin**^**c**^	**2,3-butanediol**^**b**^	**Hydroxyacetone**^**b**^
D-glucose (Glu)	37.2	1.4	4.1	0.0	0.0	3.5	trace	0.4	trace
D-mannose (Man)	25.3	11.3	2.3	0.0^d^	0.2	2.2	0.1	0.0	1.7
D-arabinose (D-Ara)	32.1	2.2	1.8	23.0^a^	0.1	3.0	0.0^d^	0.0	0.0
L-arabinose (L-Ara)	27.0	12.3	6.1	0.0	0.1	1.7	0.1	0.0	3.8
D-xylose (Xyl)	34.6	6.4	2.1	0.0^d^	0.0	3.3	0.1	0.0	3.5
D-fucose (D-Fuc)	36.6	11.0	3.8	0.9	2.2	9.1	0.0	0.0	0.0
L-fucose (L-Fuc)	33.5	8.0	6.7	0.3	ND^e^	7.1	0.0	0.0	trace

The unit of the metabolites is mM/OD_600._

^a^ The concentrations of major metabolites were determined using HPLC.

^b^ Estimated concentrations were based on manual spectral peak deconvolution, rather than by automated (Chenomx) method.

^c^ Acetoin could be quantified by the Chenomx 7.61 software, not the Chenomx 7.51 version.

^d^ The metabolites could be detected in the continuous cultures, not in the batch cultures.

^e^ Concentration could not be accurately determined.

### Metabolite coverage of NMR analysis

**Figure 1:**
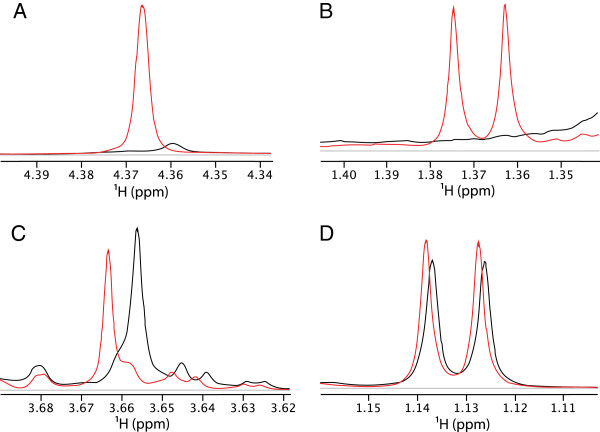
**Spectra illustrating selected identified compounds in cell extracts and supernatants.** Black line represents extract spectral line, red represents supernatant spectral line. Panel **A** (mannose substrate) shows peak for hydroxyacetone (singlet at 4.368ppm, not in Chenomx database) present in supernatant (red) and not in extract (black). Panel **B** (L-arabinose substrate) shows methyl doublet for acetoin (1.368 ppm) present in supernatant (red) but not cell extract (black). Panel **C** (D-arabinose substrate) shows ethylene glycol present in both supernatant (red) and extract (black), and Panel **D** (L-fucose substrate) shows propylene glycol present in both supernatant (red) and extract (black).

A complete list of compound assignments is provided in the supplemental material (Additional file 1). Selected identifications and quantifications of specific metabolites illustrating differences in supernatant and cell extracts are shown in Figure 1. Observed ^1^H and ^13^C chemical shifts and corresponding assignments for major metabolites are shown in Table 1. Multiplicities in 1-D ^1^H spectra and observation of the expected cross peaks in 2-D ^1^H-^1^H COSY (COrrelation SpectroscopY), ^1^H-^13^C HSQC (Heteronuclear Single Quantum Coherence), and ^1^H-^13^C HMBC (Heteronuclear Multiple Bond Correlation) spectra confirmed these assignments. All shifts are in agreement with expected and previously reported values.

### Identification of novel metabolites

**Figure 2:**
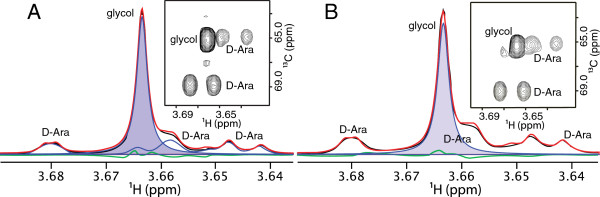
**Spectra illustrating identification and confirmation of ethylene glycol in cultures utilizing D-arabinose as substrate.** In both panels, the black line indicates the original experimental data, the red line indicates the sum of the fits of individual spectral components (arabinose and ethylene glycol) identified by Chenomx, and the green line indicates the difference spectrum of the experimental and sum-fit spectra. Panel **A** shows the spectrum of the uninoculated media with (black line) and without (unshaded blue line) ethylene glycol, and the shaded blue area represents the fit of the ethylene glycol peak after addition to media; the inset shows 2-D HSQC confirming the peak assignments. Panel **B** shows ethylene glycol present in the culture supernatant of *C. saccharolyticus* grown on D-arabinose using the same color scheme, absent the uninoculated media spectrum used in panel **A**; the inset shows the 2-D HSQC spectrum.

**Figure 3:**
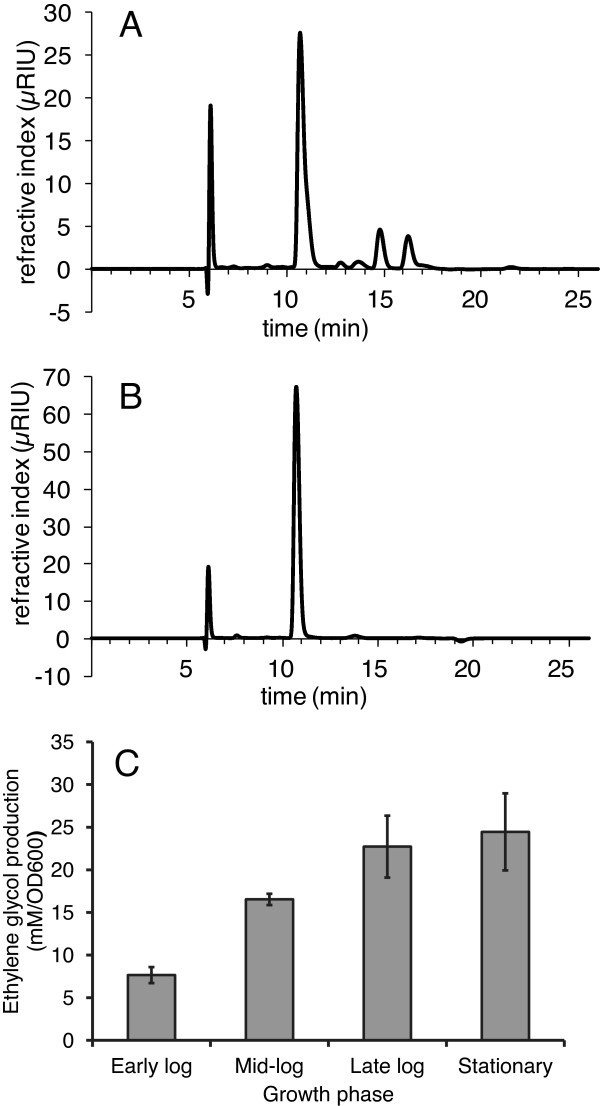
**HPLC analysis of ethylene glycol in culture media and ethylene glycol production in different growth phases.** Panel **A**: a representative chromatogram of medium composition obtained from the *C. saccharolyticus* DSM 8903 strain grown in BA medium supplemented with 10 g/L D-arabinose. D-arabinose was eluted at 10.70 min, lactate at 12.77 min, glycerol at 13.69 min, acetate at 14.81 min, ethylene glycol at 16.25 min, and ethanol at 21.50 min. Panel **B**: BA medium supplemented with 10 g/L D-arabinose. Panel **C**: *C. saccharolyticus* produces the highest concentrations of ethylene glycol during the late log phase. Values for ethylene glycol concentrations in culture media are from three independent experiments (n=3). Error bars represent standard deviations from the means.

Acetate and lactate were the most abundant products during growth on each substrate, and small quantities of ethanol and glycerol were found in all cultures. In addition to acetate, lactate, glycerol, and ethanol, culture supernatants from growths on D-glucose also contained small amounts of the C_4_ compounds acetoin and 2,3-butanediol (Table 2). Culture supernatants from cells grown on D-arabinose contained a significant concentration (~ 23 mM/OD_600_) of ethylene glycol, an uncommon fermentation product. Because ethylene glycol has single ^1^H and ^13^C chemical shifts, and because these shifts are nearly coincident with those of one of the H5 protons and C5 in one of the D-arabinose anomers, the assignment suggested by the Chenomx software was confirmed by spiking samples of uninoculated media and culture supernatant with ethylene glycol and observing the expected increase in intensity of the putative ethylene glycol peak in the HSQC spectrum (Figure 2). According to our confirmatory HPLC analysis, *C. saccharolyticus* produced the highest concentrations of ethylene glycol during late log phase (Figure 3). Although the ethylene glycol concentration seems slightly higher in the stationary phase compared to the late log phase (Figure 3), the difference is not statistically significant (P=0.55 using *t*-test, n=3). Propylene glycol was further observed (up to 5 mM with longer growth time) in both supernatants and extracts from cultures grown on L-fucose.

**Figure 4:**
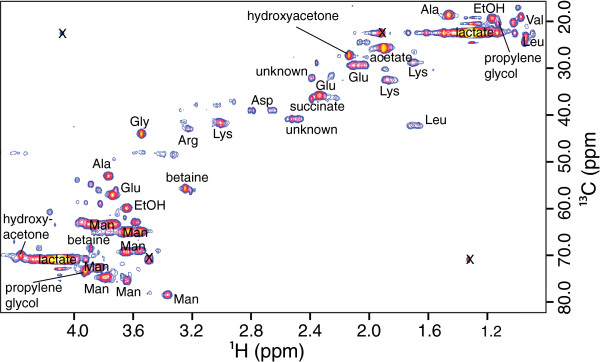
**2-D**^**1**^**H-**^**13 **^**C HSQC of supernatant from culture grown on D-mannose, annotated with compound identifications based on chemical shifts.** Standard three letter amino acid and monosaccharide abbreviations are used. Artifact peaks are marked with X. Two unassigned peaks are indicated.

Hydroxyacetone and 2,3-butanediol were not identified in our primary 1-D ^1^H NMR screen using the Chenomx software, because its library does not contain their NMR spectra. Instead, these compounds were identified in supernatant mixtures after 2-D COSY, HSQC, and HMBC experiments were conducted to identify the unassigned features in the 1-D spectra. A representative HSQC spectrum with assignments for a mannose culture supernatant is shown in Figure 4. Identified compound concentrations were estimated by using spectral deconvolution to determine peak areas in 1-D ^1^H spectra, then comparing the areas to peaks of known concentration. Compound identifications were also confirmed by comparison with prepared standards of individual compounds. In particular, the 2,3-butanediol was shown by comparison to standards to be (2R,3R) and/or (2S,3S)-butanediol, rather than the *meso* (2R,3S)-butanediol diastereomer. We did not assay optical activity of the butanediol to determine what proportions of the two enantiomers were produced. Bacterial production of all three stereoisomers has been reported in various microorganisms [16]. *C. saccharolyticus* is predicted to be capable of producing 1,2-propanediol from fermentation of L-fucose on the basis of the presence of a predicted lactaldehyde reductase gene [4]; our results confirm this prediction. In addition to these major products, we also observed production of the amino acids alanine and glycine. Although alanine and glycine are present in the culture medium, concentrations of both amino acids in the supernatant were significantly greater compared to their concentrations in the uninoculated growth medium. Yeast extract in the growth medium provides amino acids; alanine was determined to be ca. 0.6 mM and glycine was ca. 0.3 mM. Moreover, ^13^C enrichment in alanine in the supernantant arising from [1-^13^C]-glucose in cultures supplemented with this labeled substrate proves that significant alanine production and secretion occurred during these culture growths. None of the major fermentation products (Table 2) were defined components of the growth medium, and we confirmed that none were introduced to the medium by addition of yeast extract.

In summary, *C. saccharolyticus* was grown on BA media supplemented with different monosaccharide substrates to develop preliminary metabolite profiles for metabolic reconstruction and identify unknown metabolites. These screening experiments revealed several fermentation products that to our knowledge had not been observed previously in *C. saccharolyticus*: ethylene glycol, 2,3-butanediol (RR and/or SS but not *meso* stereoisomers), acetoin (likely the precursor to 2,3-butanediol), and hydroxyacetone. Of these, ethylene glycol was the most abundant (ca. 23 mM per OD_600_ in growth on 10 g/L D-arabinose during late log phase) in the culture supernatant. Formation of ethylene glycol, acetoin, and 2,3-butanediol in particular are likely not to be byproducts of non-fermentative processes, rather they are almost certainly the products of fermentative reduction of more oxidized precursors. While ethylene glycol is uncommon, acetoin and 2,3-butanediol are well-known fermentation products in some bacteria [16,17]. However, we were not able to immediately identify a candidate *C. saccharolyticus* gene for acetoin formation (presumably, acetolactate decarboxylase), though several candidate acetoin dehydrogenases (Csac_2718, 0357, 0868, or 1605) that could reduce acetoin to 2,3-butanediol have been identified in the genome [4].

*C. saccharolyticus* cells grew poorly in BA medium supplemented with 1% glucose without yeast extract. The optical density at a wavelength of 600 nm (OD_600_) is 0.069 after 48 hr incubation at 65°C; OD_600_ of cell culture grown in BA medium supplemented with 1% glucose and 0.2% yeast extract is 0.283 after 48 hr incubation at 65°C based on two independent experiments. Therefore, a richer medium, BA medium supplemented with 0.2% yeast extract was utilized.

### D-arabinose fermentation

In cells grown on D-arabinose, ethylene glycol was a major product, produced at roughly comparable levels to acetate. Ethylene glycol was not observed in significant quantities as a product of growth on any other substrate used in this study, including L-arabinose. Ethylene glycol production from fermentative anaerobic carbohydrate metabolism appears to be unusual. The likely precursor would be glycolaldehyde, which could be reduced by an alcohol dehydrogenase coded in the *C. saccharolyticus* genome, such as Csac_0622 [4]. The catabolic route of D-arabinose as predicted from the genome [4] does not provide a straightforward route to glycolaldehyde via the non-oxidative pentose phosphate pathway. Indeed, the predicted pathway for D-arabinose catabolism via D-ribulose does not identify a candidate gene for D-ribulokinase that would yield D-ribulose-5-phosphate, the precursor to D-xylulose-5-phosphate and/or D-ribose-5-phosphate [4]. Furthermore, growth on D-xylose, which is also metabolized via the non-oxidative pentose phosphate pathway and would be expected to yield D-xylulose-5-phosphate, produces only very low levels (less than 200 μM) of ethylene glycol. Instead, a proposed direct route to ethylene glycol from D-arabinose that has been demonstrated in *E. coli* mutants utilizes the L-fucose pathway, a pathway which appears also to be present in *C. saccharolyticus*[18]. Indeed, L-fucose isomerase (Csac_1339) is annotated as D-arabinose isomerase [4] and the purified recombinant protein has activity with D-arabinose substrate [19]. In this proposed route, catabolism of the pentose D-arabinose by the (hexose utilizing) L-fucose pathway, could produce two-carbon glycolaldehyde in place of three-carbon lactaldehyde, and the glycoaldehyde could then be reduced to ethylene glycol. The stereochemical configurations at C2, C3, and C4 in D-arabinose and L-fucose are identical, as has previously been noted [18,19], and the cyclic pyranose form of D-arabinose is identical to that of L-fucose with the exception of the C6 methyl group in L-fucose which is replaced with a hydrogen atom in D-arabinose.

### D-xylose and L-arabinose fermentation

The mixture of fermentation products resulting from growth on D-xylose was somewhat similar to that from growth on L-arabinose. Small amounts of ethanol and glycerol were produced from growth on D-xylose and L-arabinose as well (Table 2). These similarities may indicate that D-xylose and L-arabinose utilization pathways merge at D-xylulose-5-phosphate. No 2,3-butanediol was produced in D-xylose or L-arabinose fermentation; however, acetoin and hydroxyacetone were observed in minor amounts in cultures grown on L-arabinose (see Table 2).

### D-mannose fermentation

Growth of *C. saccharolyticus* on D-mannose, the C-2 epimer of glucose, produced more lactate compared to growth on glucose (Table 2). More lactate production in cultures grown on D-mannose versus that in D-glucose grown cultures (~ 8 fold) is not easily explained. Willquist and van Niel utilized kinetic analysis to determine that *C. saccharolyticus* lactate dehydrogenase (LDH) activity is regulated through competitive inhibition by pyrophosphate and NAD and allosteric activation by fructose-1,6-bisphosphate, ATP and ADP; the authors furthermore concluded that activation of LDH by ATP indicated that *C. saccharolyticus* utilizes LDH as a means to adjust ATP and NADH production [8]. Perhaps notable is the observation of VanFossen et al. that substantial differences (≥ 2-fold change in abundance in 353 ORFs) in the *C. saccharolyticus* transcriptome were seen when cells utilizing either glucose or mannose were compared, while far fewer differences were seen when cells utilizing galactose or glucose were compared (32 ORFs with ≥ 2-fold change) [5].

Hydroxyacetone was identified in mannose-fed cultures as a minor (approximately 1.7 mM per OD_600_) product. This product was also observed in L-arabinose and D-xylose cultures (see Table 2). It is not clear what hydroxyacetone production implies about D-mannose, L-arabinose and D-xylose metabolism in *C. saccharolyticus*, though it could be the result of nonspecific enzymatic activity on substrates such as propylene glycol or methylglyoxal. In methanogenic archaea (*Methanococcus jannaschii*), reduction of methylglyoxal was shown to produce both lactaldehyde and hydroxyacetone [20]. Methylglyoxal is a central metabolite in the synthesis of aromatic amino acids in *M. jannaschii*. Alternatively, hydroxyacetone may be produced in *M. jannaschii* by condensation of pyruvate with formaldehyde with loss of CO_2_[20] though this route is unlikely in *C. saccharolyticus*.

Although non-specific enzyme activity may account for production of metabolites such as hydroxyacetone and acetoin, if these compounds are products of non-specific reactions on common metabolic intermediate precursors, we might expect to see them across all conditions. Since hydroxyacetone was identified from D-mannose, L-arabinose, and D-xylose cultures and acetoin was identified from L-arabinose, D-arabinose, D-glucose, and D-xylose cultures, it suggests that these metabolites are products of specific enzyme reactions on specific substrates.

### D-glucose fermentation

For cultures grown on glucose, ethanol (around 4.1 mM per OD_600_) and lactate (around 1.4 mM per OD_600_) were present along with the most abundant metabolite, acetate (around 37 mM per OD_600_). The novel metabolites 2,3-butanediol (2R,3R and/or 2S,3S stereoisomers) and acetoin were present at lower concentrations in the culture supernatant. We did not observe diacetyl, a possible precursor to acetoin through a non-enzymatic oxidative decarboxylation of acetolactate that is unlikely in anaerobic conditions. Butanediol fermentation is common in the *Gammaproteobacteria* and is known in some *Firmicutes* genera, both in the *Clostridia* and *Bacilli* classes, but has not been reported in *C. saccharolyticus*. Indeed, *Klebsiella pneumoniae* and *Bacillus polymyxa* have been discussed as potential industrial scale producers of 2,3-butanediol, utilizing a mixed acid fermentation pathway whose other end-products include ethanol, acetate, lactate, formate, and succinate [16]. In these organisms, formation of 2,3-butanediol begins with condensation of two pyruvates by acetolactate synthetase to yield acetolactate and CO_2_[21,22]. Acetohydroxyacid synthetases are common, due to their role in biosynthesis of L-valine, L-leucine and L-isoleucine. The *C. saccharolyticus* genome has genes for two such enzymes (Csac_0837 and Csac_1142/1143) annotated as acetolactate synthases. Under anaerobic conditions, decarboxylation of acetolactate by acetolactate decarboxylase produces acetoin [22]. Acetoin, typically the precursor of 2,3-butanediol, can be reduced in a reversible reaction catalyzed by acetoin reductase, which also catalyzes the irreversible reduction of diacetyl to acetoin [16]. However, we could not identify an acetolactate decarboxylase in the *C. saccharolyticus* genome.

An alternate route to 2,3-butanediol from acetoin via diacetyl and acetylacetoin has also been suggested in which diacetyl is acetylated and reduced to yield acetylbutanediol which is then hydrolyzed to 2,3-butanediol and acetate [23]. We cannot rule out that. *C. saccharolyticus* may use this alternate route or some variation of the typical route to 2,3-butanediol, or it may have a novel or atypical acetoin reductase that cannot be identified by sequence comparisons. In any case, identification of the genes essential for 2,3-butanediol formation and determination of the stereochemistry of this mechanism will be important goals going forward.

### D- and L-fucose metabolism

A limited batch culture investigation of growth on the deoxyhexoses L-fucose and D-fucose was conducted to confirm genome-based predictions about their metabolism [4], particularly as it relates to D-arabinose metabolism (*vide infra*). These substrates did not support robust growth in our lab (highest OD_600_ of L-fucose culture is 0.10; highest OD_600_ of D-fucose culture is 0.08 according to three independent batch cultures), though growth on this substrate has been reported previously [1] and was predicted on the basis of the presence of two genes in the *C. saccharolyticus* genome, Csac_1340 and Csac_1339 that code for putative α-L-fucosidase and L-fucose isomerase enzymes. The predicted pathway results in 1,2-propanediol formation, in agreement with our observations. If the same pathway were used to support metabolism of the pentose D-arabinose, ethylene glycol would be the expected fermentation product. This expectation was confirmed in our results, and indeed appears to be more facile than conversion of L-fucose to 1,2-propanediol. As with L-fucose, growth on D-fucose was slow, requiring 48 hr to reach an OD of approximately 0.08. The route of D-fucose utilization in *C. saccharolyticus* is currently under investigation.

## Conclusions

Our approach using 1-D ^1^H and ^13^C NMR spectroscopy to characterize product mixtures from monosaccharide fermentation by *C. saccharolyticus* identified numerous components in culture supernatants that were not present in the growth medium prior to inoculation. Components that could not be assigned from 1-D spectra because they were not present in our spectral databases were assigned and identified with 2-D NMR experiments and confirmed by comparison to authentic standards. This approach has particular advantages over other methods of analyzing products of microbial cell culture. Minimal sample manipulation is required, no derivatization is necessary, and information useful for identification of novel metabolites is obtained. The main disadvantage, of course, is the inherent low sensitivity of NMR spectroscopy, such that minor metabolite components that might be of interest may not be observable.

Together, the results suggest that *C. saccharolyticus*, already of substantial interest due to its potential for biological ethanol and hydrogen production, has further metabolic potential for production of the higher molecular weight compounds acetoin and 2,3-butanediol, as well as glycerol, hydroxyacetone, and the uncommon fermentation product ethylene glycol. In addition to these alcohols, production of acetate and lactate was observed. Formation of reduced, non-acidic, fermentation products may be a built-in mechanism for bacteria to mitigate excess acetate or lactate formation, which could decrease the pH of the growth medium to a point that may not be beneficial to the organism. Conceivably, metabolic pathways could be engineered to divert carbon away from two-carbon products such as ethanol and acetate and towards reduced three and four carbon products such as glycerol or butanediol. The findings presented here seem to suggest *C. saccharolyticus* has multiple routes available by which this strategy could be implemented. Furthermore our finding that *C. saccharolyticus* produces substantial amounts of ethylene glycol during growth on D-arabinose, apparently from glycolaldehye by the L-fucose pathway (which perhaps should be called the L-fucose/D-arabinose pathway) adds this reduced fermentation product that may be of interest in industrial biotechnology as a product of lignocellulosic biomass.

## Materials and methods

### Reagents

Components of the growth medium were obtained from Sigma (St. Louis, MO) and used without further purification. Carbon-13 labeled glucose ([1-^13^C]-D-glucose) was obtained from Cambridge Isotope Laboratories (Andover, MA).

### Bacterial strain and growth conditions

*C. saccharolyticus* DSM 8903 was obtained from the Deutsche Sammlung von Mikroorganismen und Zellkulturen (DSMZ). *C. saccharolyticus* was grown in the anaerobic BA medium. The BA medium composition has been described previously [24,25]. BA medium contains 18.7 mM NH_4_Cl, 1.71 mM NaCl, 0.5 mM MgCl_2_, 0.34 mM CaCl_2_, 1.8 mM K_2_HPO_4_, 2 μM resazurin, 0.81 μM boric acid, 0.37 μM ZnCl_2_, 0.22 μM CuCl_2_, 0.25 μM MnCl_2_, 0.040 μM (NH4)_6_Mo_7_O_24_, 0.37 μM AlCl_3_, 0.21 μM CoCl_2_, 0.39 μM NiCl_2_, 1.34 μM EDTA, 0.25 μM Na_2_SeO_3_, and 9.83 μM FeCl_2_. The vitamin solution and cysteine used previously in BA medium were omitted, and instead the medium was supplemented with 2 g/L yeast extract and the appropriate monosaccharide substrate at a concentration of 10 g/L. Media were made anaerobic by flushing with N_2_/CO_2_ (80/20, v/v).

To compare the growth and the metabolite levels across the different monosaccharides tested, *C. saccharolyticus* was grown on D-glucose, D-mannose, D-xylose, L-arabinose, D-arabinose, L-fucose, and D-fucose in batch cultures. The growth was further tested in continuous culture with L-arabinose, D-arabinose, D-mannose and D-xylose as substrate. Batch cultivation experiments were performed with a culture volume of 20 ml in an airtight flask at 65°C. Continuous cultivation was performed 60°C in an INFORS HT Multifors (Basel, Switzerland) bench top bioreactor at a constant working volume of 0.5 L with stirring at 100 rpm. The pH was controlled at 7.0 by automatic addition of NaOH to the vessel. Fresh media was added at a rate of 0.12 ml/min. The software Iris V5 was used to control the bioreactor and analyze and archive the data. The BA medium in the vessel of the bioreactor was inoculated with 1% (v/v) of seed culture in the exponential growth phase. A culture was considered to have reached steady state when the bacterial culture remained at a constant optical density (OD) at wavelength of 600 nm, acetate or lactate production rates remained constant (monitored by HPLC), and at least four volume changes (2 L) had occurred. Samples were collected at steady states for the determination of intracellular and extracellular metabolites.

### Sample preparation for NMR analysis

To prepare samples of extracellular metabolites for identification and quantification by NMR spectroscopy, 1 ml volumes were removed from the culture and centrifuged at 14000 rpm (20,800 rcf) for 5 minutes at 4°C. The resulting supernatant samples were not filtered. For each sample, a supernatant volume of 540 μl was mixed with 60 μl of D_2_O solution containing 5 mM DSS-d6 (2,2 dimethyl 2-silapentane-d6 5-sulfonate, sodium salt) for chemical shift referencing and 0.2% (w/v) sodium azide as a microbiocide. To determine intracellular metabolites, a sample of 10 ml was harvested and centrifuged. The cell pellet was mixed with ice-cold chloroform/methanol (2:1, v/v) solution and subjected to three cycles of freeze/thaw using liquid nitrogen. Following centrifugation, the sample separated into three layers (aqueous, cell debris, and organic). The entire upper layer (aqueous MeOH) was removed and evaporated under vacuum centrifugation in a Savant SpeedVac concentrator (Thermo Fisher). For each sample, the resultant dried extract was mixed with 300 μl of D_2_O solution containing 0.5 mM DSS-d6 for chemical shift referencing and 0.2% (w/v) sodium azide as a microbiocide.

### NMR spectroscopy

Extracellular metabolite samples were placed in 5 mm 535-PP NMR tubes (Wilmad), and intracellular samples were placed in 5 mm Shigemi NMR tubes. All NMR spectra were collected at 25°C on a 600 MHz Agilent (Varian) NMR System equipped with a salt-tolerant 5 mm HCN coldprobe with cold carbon preamplifier for higher sensitivity in ^13^C-observe experiments. Samples contained 0.5 mM DSS-d6 for chemical shift referencing and as an internal standard for quantification. For Chenomx analysis, 1-D NOESY spectra were collected using the Varian tnnoesy pulse sequence with 12 ppm spectral width, acquisition time of 4 seconds, mixing time of 100 milliseconds, relaxation delay of 1 s, and 128 scans. Direct observe 1-D ^13^C spectra were collected using a 224 ppm spectral width, a tip angle of 45°, a relaxation delay of 3 seconds, and WALTZ proton decoupling during the acquisition time of 1.3 seconds. Two-dimensional ^1^H-^1^H magnitude COSY and ^1^H-^13^C HSQC and HMBC experiments were collected using Varian gCOSY, gHSQC, and gHMBC pulse sequences with ^1^H spectral width of 12 ppm and ^13^C spectral widths of 170 ppm (HSQC) or 240 ppm (HMBC), with an acquisition time of 200 milliseconds (1445 complex points), 128 complex points in the indirect dimension for HSQC and HMBC and 512 for COSY experiments, 128 transients, 1s recycle delay, and adiabatic WURST decoupling (Varian W40_Coldprobe) during acquisition in the HSQC experiment.

### NMR data analysis

One-dimensional ^1^H spectra were processed and analyzed with Chenomx software version 7.61 (Edmonton, AB) with 0.5 Hz line broadening and automatic baseline correction. Quantification of 1-D spectra relied on comparison of peak areas in compound peak clusters to the concentration standard of 0.5 mM DSS-d6 (2,2 dimethyl 2-silapentane-d6 5-sulfonate, sodium salt) in the Chenomx database. For analysis of extracellular (secreted) metabolites in particular, essentially no manipulation of the sample is required aside from addition of a small amount of reference solvent containing D_2_O for locking the spectrometer and DSS-d6 for referencing the spectra. Spectral features not assigned by Chenomx were further characterized using 2-D NMR experiments. Compounds were identified by *de novo* assignment of spin systems in ^1^H-^13^C HSQC and HMBC, and ^1^H-^1^H magnitude COSY experiments, and identifications were confirmed by spectral comparison to authentic compounds. Concentrations of novel metabolites were estimated using spectral deconvolution routines within the spectrometer software (Varian/Agilent VnmrJ). Two-dimensional NMR data was processed using Felix (FelixNMR, Inc). The 2-D heteronuclear experiments were processed with time-domain convolution of the water resonance followed by apodization with a 90 degree shifted sinebell window matched to the entire FID and zero-filling to twice the number of real points. The same apodization and zero-filling were applied to the indirect dimension after linear prediction of 30% more real points. Magnitude COSY spectra were processed in both dimensions with 10 degree-shifted squared sinebell apodization and zero-filling following time-domain solvent deconvolution of the acquired data.

### HPLC analysis

To analyze extracellular metabolites using HPLC, 1 mL samples were removed from the *C. saccharolyticus* DSM 8903 culture and centrifuged at 14000 rpm (20,800 rcf) for 5 minutes at 4°C. The resulting supernatants were filtered before HPLC analysis. Samples were analyzed using an Ultimate 3000 HPLC system (Dionex) consisting of a pump, an autosampler and a column compartment. The column was a 300 mm × 7.8 mm Aminex HPX-87H column (Bio-Rad) and the column temperature was 60°C. The eluent was 4 mM sulfuric acid solution. The flow rate was maintained at 0.6 ml/min. The HPLC system was equipped with a refractive index detector (RI-101, Shodex). Chromeleon 7 software was used to integrate the peaks and quantify the metabolites.

### Bioinformatics

Candidate genes for acetoin and butanediol production were searched using PSI-BLAST to find sequence homologues of annotated acetolactate synthase, acetolactate decarboxylase, and acetoin dehydrogenase genes from other bacteria.

## Abbreviations

COSY: COrrelation SpectroscopY; ED: Entner-Doudoroff; EM: Embden-Meyerhof; HSQC: Heteronuclear Single Quantum Coherence spectroscopy; HMBC: Heteronuclear Multiple Bond Correlation spectroscopy; NMR: Nuclear Magnetic Resonance

## Competing interests

The authors declare they have no competing interests.

## Authors’ contributions

NI designed the NMR studies, collected and analyzed NMR data, and wrote the manuscript. JX designed and conducted batch and continuous culture studies, prepared samples, collected HPLC data, and revised the manuscript. JR participated in continuous culture studies and sample preparation. JC analyzed NMR data and wrote the manuscript. BK led design of the *C. saccharolyticus* studies and revised the manuscript. All authors have read and approved the manuscript.

## Supplementary Material

Additional file 1 Metabolite concentrations determined using proton NMR spectroscopy (μM). Click here for file
