# Supplementary material for: Novel monosaccharide fermentation products in Caldicellulosiruptor saccharolyticus identified using NMR spectroscopy
Source: Biotechnol Biofuels. 2013 Apr 3;6:47. doi: 10.1186/1754-6834-6-47 (PMC3637100; doi:10.1186/1754-6834-6-47)
Supplement: Additional file 1 — Metabolite concentrations determined using proton NMR spectroscopy (μM). [file 1754-6834-6-47-S1.docx]

| Metabolite concentrations determined using proton NMR spectroscopy (µM) | | | | | | |  |  |  |  |
| --- | --- | --- | --- | --- | --- | --- | --- | --- | --- | --- |
|  | D-xylose, 1%, continuous | D-glucose, 1%, batch^ | D-mannose, 1%, continuous | L-arabinose, 1%, batch^ | D-arabinose, 1%, batch^ | L-arabinose, 1%, continuous | D-arabinose, 1%, continuous | D-fucose, 1%, batch^ | D-fucose, 1%, batch^ | L-fucose, 1%, batch^ |
| 1,3-Dihydroxyacetone | 29.7 | 17.5 | 16 | 48.9 | 20.9 | 43.7 | 10.8 | 5.7 | 5.7 | 11 |
| 2-Oxoglutarate | 0 | 0 | 0 | 0 | 0 | 0 | 0 | 0 | 0 | 0 |
| 4-Hydroxyphenylacetate | 0 | 0 | 0 | 0 | 0 | 0 | 0 | 0 | 0 | 0 |
| Acetate | 3586.6 | 11163.9 | 5854.7 | 8680.7 | 4772.4 | 9892.3 | 16530.2 | 1585.4 | 1706.4 | 1927.7 |
| Acetoin | 32.2 | 43.6 | 0 | 636.7 | 0 | 892.6 | 106.8 | 0 | 0 | 0 |
| Acetone | 23.9 | 26.2 | 27 | 400.9 | 0 | 574.1 | 66.3 | 0 | 0 | 0 |
| Adenine | 12.3 | 19.2 | 2.2 | 17 | 26.8 | 14.5 | 22.3 | 20.1 | 20.1 | 10.7 |
| Adenosine | 4.3 | 5.9 | 6.6 | 5.4 | 4 | 5.4 | 6.6 | 4.6 | 4.6 | 12.6 |
| Alanine | 1083.2 | 1267.8 | 1161.5 | 1971.5 | 790 | 2477.6 | 1765 | 683 | 681 | 792.2 |
| Arabinose | 0 | 0 | 0 | 32159.8 | 44954.5 | 16722.1 | 27579.3 | 0 | 0 | 0 |
| Arginine | 85.8 | 119.2 | 154.3 | 109.2 | 109.2 | 83.1 | 133 | 107.7 | 107.7 | 119.6 |
| Asparagine | 62.2 | 167.8 | 121.3 | 139.8 | 125.4 | 139.8 | 150.1 | 134.5 | 134.5 | 113.2 |
| Aspartate | 351.8 | 315.9 | 260.6 | 277.1 | 263.9 | 280.6 | 304 | 247.4 | 267.4 | 279.9 |
| Betaine | 143.1 | 143.1 | 130.4 | 139.3 | 139.3 | 142.8 | 150.3 | 135.9 | 135.9 | 144.4 |
| Cysteine | 0 | 0 | 0 | 0 | 0 | 0 | 0 | 0 | 0 | 0 |
| Cystine | 0 | 0 | 0 | 0 | 0 | 0 | 0 | 0 | 0 | 0 |
| DSS-d6 (Chemical Shape Indicator) | 495.3 | 495.3 | 493.5 | 495.3 | 495.3 | 495.3 | 495.3 | 495.3 | 495.3 | 495.3 |
| Ethanol | 936.3 | 1184.2 | 1229.9 | 2369.1 | 407.1 | 3471.1 | 1721.9 | 194.4 | 126.6 | 385 |
| Ethylene glycol | 166.6 | 0 | 206.8 | 0 | 1211.7 | 0 | 14197.9 | 78.2 | 78.2 | 78.2 |
| Formate | 192.5 | 60.1 | 123.6 | 64.3 | 96.3 | 183.4 | 161.6 | 89.7 | 89.7 | 137.9 |
| Fucose | 0 | 0 | 0 | 0 | 0 | 0 | 0 | 42474.3 | 42474.3 | 42934.7 |
| Fumarate | 5.4 | 7.5 | 5 | 18 | 1.6 | 34.4 | 15.8 | 1.4 | 1.2 | 2.4 |
| Glucose | 0 | 50419.2 | 0 | 0 | 0 | 0 | 0 | 0 | 0 | 0 |
| Glutamate | 739.1 | 828.1 | 775.8 | 739.1 | 696 | 967 | 773.1 | 739.1 | 663.2 | 676.6 |
| Glutamine | 0 | 0 | 19.3 | 0 | 0 | 0 | 0 | 0 | 0 | 0 |
| Glycerol | 862.3 | 621.5 | 704.6 | 674.4 | 674.4 | 783.7 | 674.4 | 409.8 | 409.8 | 409.8 |
| Glycine | 1032.2 | 679.1 | 1036.1 | 1630.3 | 432.2 | 2190.3 | 1015 | 308.1 | 309.2 | 552.2 |
| Glycolate | 18.5 | 15.2 | 34.3 | 18.5 | 0 | 0 | 0 | 18.5 | 18.5 | 18.5 |
| Guanosine | 6.7 | 6.7 | 9 | 5.3 | 5.3 | 5.3 | 7.1 | 7 | 5.9 | 6.7 |
| Histidine | 44.1 | 78 | 0 | 59.4 | 58.9 | 44.4 | 46.8 | 35.4 | 39.6 | 41.4 |
| Imidazole | 0 | 0 | 0 | 0 | 0 | 0 | 0 | 0 | 0 | 0 |
| Isoleucine | 205.7 | 305.1 | 92.3 | 304.9 | 252.5 | 281.5 | 230.7 | 253.7 | 252.1 | 296.3 |
| Lactate | 36106.9 | 4146.3 | 67917.6 | 6341.8 | 487.6 | 27232.4 | 6782.6 | 419.9 | 444.9 | 462.1 |
| Leucine | 287.5 | 443 | 186.6 | 335 | 335 | 278.9 | 335 | 330.4 | 330.4 | 388.9 |
| Lysine | 242.5 | 300.5 | 209.6 | 313.2 | 274.1 | 343 | 274.1 | 218.2 | 218.2 | 273.2 |
| Maleate | 1.4 | 1.4 | 1.8 | 1.2 | 1.1 | 1.2 | 1.5 | 1.3 | 1.3 | 1.1 |
| Mannose | 0 | 0 | 1220.8 | 0 | 0 | 0 | 0 | 0 | 0 | 0 |
| Methanol | 20.1 | 11.8 | 18.6 | 10.3 | 13.7 | 35.2 | 49.6 | 4.2 | 4.2 | 1.7 |
| Methionine | 45.4 | 79.5 | 26.2 | 71.9 | 70.4 | 61 | 70.4 | 57.2 | 65.3 | 42.1 |
| Nicotinate | 11 | 3.6 | 15.1 | 5.4 | 1.8 | 13.1 | 8.5 | 0 | 0 | 0 |
| Phenylacetate | 0 | 0 | 0 | 0 | 0 | 0 | 0 | 0 | 0 | 0 |
| Phenylalanine | 137.5 | 209.3 | 98.6 | 165.2 | 180 | 131.6 | 162.3 | 177.9 | 178.7 | 191.5 |
| Proline | 156.9 | 156.9 | 175.3 | 128.9 | 97.4 | 128.9 | 97.4 | 82 | 82 | 124.2 |
| Propylene glycol | 0 | 0 | 151.6 | 0 | 0 | 102.7 | 87.8 | 129.4 | 120.9 | 540.7 |
| Pyroglutamate | 275 | 270.6 | 243.9 | 248.4 | 225.7 | 248.4 | 250.1 | 248.4 | 248.4 | 248.4 |
| Pyruvate | 151.7 | 895.2 | 213 | 1143.4 | 67.8 | 352.8 | 424.7 | 23.6 | 27 | 54.1 |
| Serine | 229.2 | 305.9 | 0 | 229.2 | 229.2 | 229.2 | 0 | 229.2 | 229.2 | 229.2 |
| Succinate | 161.4 | 180.4 | 151.6 | 147.3 | 145.1 | 173.5 | 168 | 134.1 | 131.1 | 146.5 |
| Threonine | 185.6 | 188.1 | 0 | 227.7 | 187.5 | 259.2 | 171.1 | 176.2 | 176.2 | 232.6 |
| Tryptophan | 35 | 50 | 23.3 | 40.8 | 36.3 | 31.8 | 36.3 | 35 | 36.9 | 20.7 |
| Tyramine | 0 | 0 | 0 | 0 | 0 | 0 | 0 | 0 | 0 | 0 |
| Tyrosine | 161.8 | 214.2 | 142.8 | 181.1 | 170.9 | 181.1 | 166.4 | 173.5 | 170.7 | 181.3 |
| Uracil | 2.2 | 7 | 0.9 | 2.2 | 7.7 | 2.2 | 1.9 | 18.5 | 18.5 | 21.2 |
| Uridine | 7.7 | 4 | 9.8 | 3.4 | 3.4 | 1.5 | 3.4 | 4.3 | 4.3 | 2.9 |
| Valine | 417.5 | 765.6 | 290.5 | 3024 | 448.7 | 2935.8 | 1011.4 | 353.1 | 365 | 469.2 |
| Xylose | 25638.5 | 0 | 0 | 0 | 0 | 0 | 0 | 0 | 0 | 0 |

|  | D-arabinose, 1%, batch# | D-fucose, 1%, batch# | D-fucose, 1%, batch# | D-glucose, 1%, batch# | D-mannose, 1%, batch# | D-xylose, 1%, batch# | L-arabinose, 1%, batch# | L-fucose, 1%, batch# | L-fucose, 1%, batch# |
| --- | --- | --- | --- | --- | --- | --- | --- | --- | --- |
| 1,3-Dihydroxyacetone | 0 | 0 | 0 | 0 | 0 | 46.9 | 34.3 | 2.6 | 3.1 |
| 2-Oxoglutarate | 0 | 0 | 0 | 0 | 0 | 0 | 0 | 0 | 0 |
| 4-Hydroxyphenylacetate | 0 | 0 | 0 | 0 | 0 | 0 | 0 | 0 | 0 |
| Acetate | 990.1 | 469.6 | 1251.4 | 2098.5 | 3092.8 | 2919.6 | 3261.8 | 213.8 | 1115.1 |
| Acetoin | 0 | 0 | 0 | 6.6 | 29.3 | 28.2 | 14.5 | 0 | 0 |
| Acetone | 0 | 0 | 0 | 0 | 0 | 0 | 0 | 0 | 0 |
| Adenine | 19.4 | 24.3 | 18.7 | 22.8 | 17.7 | 25.1 | 19.4 | 24.3 | 19.7 |
| Adenosine | 37.5 | 40.3 | 28.8 | 33.6 | 34 | 39.9 | 37.5 | 46.2 | 35.4 |
| Alanine | 758.1 | 775.5 | 775.5 | 834.2 | 1052.8 | 920.5 | 969.1 | 748.1 | 783.7 |
| Arabinose | 50161.1 | 0 | 0 | 0 | 0 | 0 | 48013.3 | 0 | 0 |
| Arginine | 165.9 | 165.9 | 165.9 | 165.9 | 165.9 | 165.9 | 165.9 | 165.9 | 165.9 |
| Asparagine | 0 | 0 | 0 | 0 | 0 | 0 | 0 | 0 | 0 |
| Aspartate | 236 | 234.7 | 234.7 | 243.9 | 251.3 | 248.5 | 257 | 240.1 | 239.3 |
| Betaine | 260.1 | 245.2 | 260.6 | 208.1 | 262.7 | 246.7 | 260.1 | 254.8 | 254.8 |
| Cysteine | 0 | 0 | 0 | 0 | 0 | 0 | 0 | 0 | 0 |
| Cystine | 0 | 0 | 0 | 0 | 0 | 0 | 0 | 0 | 0 |
| DSS-d6 (Chemical Shape Indicator) | 495.3 | 495.3 | 495.3 | 495.3 | 495.3 | 495.3 | 495.3 | 495.3 | 495.3 |
| Ethanol | 143.5 | 0 | 0 | 252 | 530.8 | 391.8 | 458.3 | 0 | 103.7 |
| Ethylene glycol | 0 | 0 | 70.1 | 0 | 0 | 0 | 0 | 0 | 23.2 |
| Formate | 94.4 | 51.8 | 62.7 | 42.9 | 42.9 | 56.4 | 61.9 | 44.7 | 44.7 |
| Fucose | 0 | 50627.9 | 50627.9 | 0 | 0 | 0 | 0 | 54994 | 52820.3 |
| Fumarate | 2.2 | 2.2 | 2.2 | 2.5 | 3.3 | 2.5 | 3 | 2.2 | 2.2 |
| Glucose | 0 | 0 | 0 | 47361.6 | 0 | 0 | 0 | 0 | 0 |
| Glutamate | 738 | 765 | 765 | 765 | 800.1 | 803.3 | 772.4 | 802.4 | 759.5 |
| Glutamine | 0 | 0 | 0 | 0 | 0 | 0 | 0 | 0 | 0 |
| Glycerol | 0 | 0 | 0 | 0 | 0 | 0 | 0 | 0 | 0 |
| Glycine | 290.6 | 357.2 | 357.2 | 415.3 | 625.4 | 601.1 | 674.5 | 357.2 | 357.2 |
| Glycolate | 0 | 0 | 0 | 0 | 0 | 0 | 0 | 0 | 0 |
| Guanosine | 0 | 0 | 0 | 0 | 0 | 0 | 0 | 0 | 0 |
| Histidine | 17.3 | 17.3 | 17.3 | 17.3 | 17.3 | 17.3 | 17.3 | 17.3 | 17.3 |
| Imidazole | 0 | 0 | 0 | 0 | 0 | 0 | 0 | 0 | 0 |
| Isoleucine | 310.3 | 324.9 | 343.4 | 317.9 | 336.5 | 359.2 | 350 | 335 | 335 |
| Lactate | 57.5 | 41.1 | 41.1 | 62.9 | 105.2 | 82 | 92.4 | 41.1 | 41.1 |
| Leucine | 458.6 | 458.6 | 458.6 | 458.6 | 458.6 | 458.6 | 458.6 | 458.6 | 458.6 |
| Lysine | 242.7 | 242.7 | 242.7 | 242.7 | 291.1 | 268 | 283 | 242.7 | 259.2 |
| Maleate | 0 | 0 | 0 | 0 | 0 | 0 | 0 | 0 | 0 |
| Mannose | 0 | 0 | 0 | 0 | 49511.7 | 0 | 0 | 0 | 0 |
| Methanol | 0 | 0 | 0 | 0 | 0 | 0 | 0 | 0 | 0 |
| Methionine | 108.2 | 108.2 | 108.2 | 97.7 | 108.5 | 109.5 | 108.2 | 108.2 | 108.2 |
| Nicotinate | 12.5 | 11.1 | 11.1 | 8.8 | 9.7 | 10.8 | 9.8 | 7.1 | 8.8 |
| Phenylacetate | 0 | 0 | 0 | 0 | 0 | 0 | 0 | 0 | 0 |
| Phenylalanine | 216.9 | 216.9 | 233.4 | 216.9 | 216.9 | 235.4 | 216.9 | 216.9 | 216.9 |
| Proline | 157.7 | 157.7 | 157.7 | 157.7 | 157.7 | 157.7 | 157.7 | 157.7 | 157.7 |
| Propylene glycol | 0 | 155.1 | 160.5 | 0 | 0 | 0 | 0 | 155.1 | 190.1 |
| Pyroglutamate | 253.7 | 253.7 | 253.7 | 227.2 | 255.7 | 242.5 | 253.7 | 238.4 | 238.4 |
| Pyruvate | 17.9 | 20.9 | 47.3 | 62.6 | 207.2 | 83 | 92.5 | 15.2 | 49.2 |
| Serine | 0 | 0 | 0 | 0 | 0 | 0 | 0 | 0 | 0 |
| Succinate | 139.9 | 134.7 | 135.8 | 134.7 | 146 | 143.7 | 148 | 137.1 | 137.1 |
| Threonine | 212 | 241.6 | 241.6 | 270.7 | 270.7 | 281.6 | 283.1 | 241.6 | 241.6 |
| Tryptophan | 52 | 52 | 52 | 54.7 | 54.7 | 58.5 | 52 | 61 | 56 |
| Tyramine | 0 | 0 | 0 | 0 | 0 | 0 | 0 | 0 | 0 |
| Tyrosine | 75.6 | 83.4 | 83.4 | 83.4 | 83.4 | 93.3 | 75.6 | 83.4 | 75.4 |
| Uracil | 12.8 | 18.6 | 18.6 | 11.2 | 6.1 | 7.2 | 6.6 | 18.6 | 18.6 |
| Uridine | 0 | 0 | 0 | 0 | 0 | 0 | 0 | 0 | 0 |
| Valine | 404.8 | 411.1 | 438.7 | 473.3 | 729 | 693.7 | 738.1 | 424.4 | 424.4 |
| Xylose | 0 | 0 | 0 | 0 | 0 | 54098.3 | 0 | 0 | 0 |

* Raw NMR data are listed. The concentrations demonstrated in the text are normalized by the dilution factor (0.9) and OD_600_ readings.

^ Samples were harvested during the mid log phase of batch cultures.

# Samples were harvested during the early log phase of batch cultures.
